# AUV Positioning Method Based on Tightly Coupled SINS/LBL for Underwater Acoustic Multipath Propagation

**DOI:** 10.3390/s16030357

**Published:** 2016-03-11

**Authors:** Tao Zhang, Hongfei Shi, Liping Chen, Yao Li, Jinwu Tong

**Affiliations:** 1 Key Laboratory of Micro-Inertial Instrument and Advanced Navigation Technology, Ministry of Education, Southeast University, Nanjing 210096, China; 220132634@seu.edu.cn (H.S.); 220132597@seu.edu.cn (L.C.); liyao@seu.edu.cn (Y.L.); 230139522@seu.edu.cn (J.T); 2 School of Instrument Science and Engineering, Southeast University, Nanjing 210096, China

**Keywords:** LBL, TDOA, tightly coupled system, fuzzy correlation peak

## Abstract

This paper researches an AUV (Autonomous Underwater Vehicle) positioning method based on SINS (Strapdown Inertial Navigation System)/LBL (Long Base Line) tightly coupled algorithm. This algorithm mainly includes SINS-assisted searching method of optimum slant-range of underwater acoustic propagation multipath, SINS/LBL tightly coupled model and multi-sensor information fusion algorithm. Fuzzy correlation peak problem of underwater LBL acoustic propagation multipath could be solved based on SINS positional information, thus improving LBL positional accuracy. Moreover, introduction of SINS-centered LBL locating information could compensate accumulative AUV position error effectively and regularly. Compared to loosely coupled algorithm, this tightly coupled algorithm can still provide accurate location information when there are fewer than four available hydrophones (or within the signal receiving range). Therefore, effective positional calibration area of tightly coupled system based on LBL array is wider and has higher reliability and fault tolerance than loosely coupled. It is more applicable to AUV positioning based on SINS/LBL.

## 1. Introduction

Autonomous underwater vehicle (AUV) is a tool that is competent for various underwater tasks, such as detection, attack, convey, salvage, *etc.* It has become an important research direction of military marine technology at home and abroad because of its wide scope, small volume, lightweight and high hidden ability. High-accuracy underwater autonomous navigation, locating and tracking technology of AUV are precondition and key to accomplish underwater tasks [1].

Currently, SINS (Strapdown Inertial Navigation System)/DVL (Doppler Velocity Log) integrated navigation is the main underwater autonomous navigation technology used in AUV in both China and foreign countries. With calibrations based on global positioning system (GPS), acoustic positioning system and magnetic compass, it can improve navigation accuracy of an AUV system effectively [2,3,4]. Acoustic positioning system can be divided into long base line, short base line and ultra-short base line systems according to distance between elements. Among them, the long base line (LBL) acoustic positioning system determines AUV position according to distance between sound source on the carrier and seabed transponder array. It has been widely used in underwater vehicles for its wide reach and high positional accuracy [5,6].

SINS/LBL integrated navigation can be divided into loosely coupled and tightly coupled in view of involved physical parameters. In some previous studies, SINS/LBL loosely coupled navigation [7,8] inhibits divergence of position error by taking difference of location information between SINS and LBL as quantity measurement of Kalman filter. Although it could increase positional accuracy of AUV effectively, the LBL could not complete the positioning when there are fewer than four available hydrophones because of carrier movement or shoal of fish. Research on SINS/LBL tightly coupled navigation is represented by Lee *et al.* [9] from South Korean Vessel and Marine Engineering Institute. He proposed an AUV integrated navigation system based on inertial sensor and pseudo-LBL acoustic transponder. It added pseudo-LBL system based on the SINS/DVL system. The pseudo-LBL system is composed of sound source on AUV and two hydrophones at seabed. The distance between sound source and each hydrophone is called pseudo-range. The differences between SINS derived pseudo-ranges and LBL measured pseudo-ranges are used as observation variables of Kalman filter. This method improves effectiveness, robustness and navigation accuracy of SINS/DVL integrated navigation system. The observed information increased by Lee *et al.* is the distance, which is obtained by the sound velocity multiplying the time of arrival (TOA) between sound source and hydrophone. Acquisition of time requires strict synchronization on time for transponder and hydrophones, which is difficult in practice. Therefore, distance measurement error is inevitable. Method based on time difference of arrival (TDOA) determines position of carrier according to time differences for sound source signal arriving to different hydrophones, which lowers requirements on time synchronization and avoids error brought by time synchronization between sound source and hydrophones. Therefore, this paper applied TDOA-based LBL acoustic system to assist SINS positioning.

TDOA is often acquired through generalized cross-correlation operation to signals receiving by two hydrophones. Since sound signal sent from sound source will make a series of refraction and reflection during the propagation process, signals received by hydrophones are actually mutual interference superposition of multipath signals, thus resulting in multiple correlation peaks with similar amplitudes. This is known as fuzzy correlation peak problem. To solve this problem, An *et al.* [10] proposed a defuzzification algorithm based on correlation peaks identification and stable correlation peaks tracking according to stability and distribution law of correlation peaks generated by different paths. This algorithm could correct TDOA estimation error brought by multipath propagation. However, the distribution of the correlation peak varies with the change of the water environment, so the proposed tracking algorithm may still cause estimation error.

To solve these problems, this paper put forward an AUV positioning system based on SINS/LBL tightly coupled algorithm of underwater acoustic propagation multipath. This system is composed of SINS, DVL, MCP, sound source and LBL acoustic positioning array. Hydrophones in LBL array receive sound source signal from AUV. A group of fuzzy correlation peaks could be gained from correlation operation of the received signals. These correlation peaks are distinguished through location information of SINS and assistance of LBL, thus getting TDOA. Later, TDOA is changed into slant-range difference. Difference between this slant-range difference and the slant-range difference calculated from SINS position is taken as the observation variables of tightly coupled system, thus correcting accumulative position error of AUV. This system uses SINS assisting to solve TDOA calculation difficulty caused by multipath propagation of underwater sound signal. Furthermore, using slant-range difference as an observation variable for filtering correction of system error, the system expands navigational correction area of AUV and makes navigation track more flexible.

In this paper, Section 2 introduces principle and structure of the system. Then, calculation method of TDOA of underwater acoustic propagation multipath and SINS/LBL tightly coupled model based on slant-range difference are introduced. Finally, some simulation experiments are implemented to prove effectiveness of the proposed algorithm.

## 2. Principle and Structure of the System

### 2.1. Principle of TDOA-Based LBL Underwater Positioning

**Figure 1:**
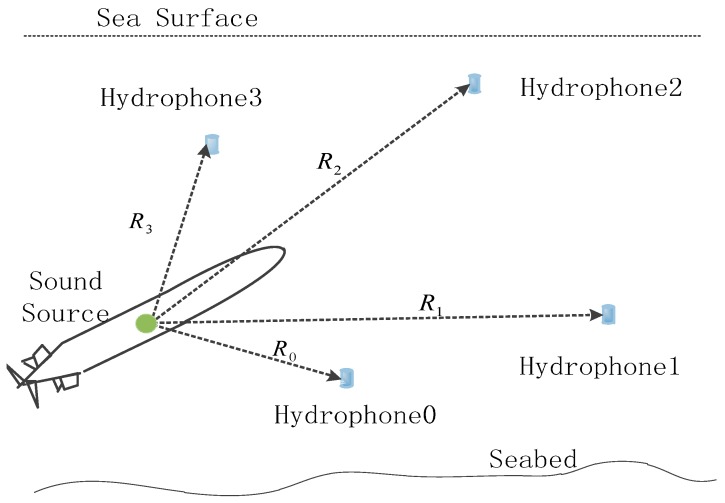
Structure of LBL (Long Base Line) system.

LBL system consists of sound source on AUV and hydrophones at seabed. Distance between hydrophones is generally 100~6000 m (Figure 1).

Common positioning algorithms in LBL acoustic positioning system include TOA positioning algorithm and TDOA positioning algorithm. TOA positioning algorithm locates AUV according to distances from sound source to hydrophones. This algorithm is easy to operate, but requires strict time synchronization between sound source and hydrophones. The TDOA positioning algorithm locates AUV according to time difference for sound signal to arrive to different hydrophones. It avoids error caused by poor time synchronization. As a result, TDOA-based LBL underwater positioning was used in this paper. Its basic principle is introduced in the following text.

Suppose AUV sends sound signal at time $t_{0}$ and hydrophone *i* receives this sound signal at time $t_{i}$. Then, propagation time from sound source to hydrophone is:

(1) $$\Delta t_{i}=t_{i}-t_{0},(i=0,1,2,3)$$

Distance between sound source and hydrophone *i* is:

(2) $$R_{i}=c\cdot \Delta t_{i}$$

where *c* is sound velocity, which is assumed a constant to simplify the analysis.

Assumed positions of hydrophones 0, 1, 2 and 3 in Figure 1 are known, which are, respectively, expressed as $P_{0}(x_{0},y_{0},z_{0})$, $P_{1}(x_{1},y_{1},z_{1})$, $P_{2}(x_{2},y_{2},z_{2})$ and $P_{3}(x_{3},y_{3},z_{3})$. Position of AUV P(x,y,z) is unknown that has to be calculated. Then, distance between sound source and hydrophone *i* could be expressed as:

(3) $$\sqrt{{\left(x-x_{i}\right)}^{2}+{\left(y-y_{i}\right)}^{2}+{\left(z-z_{i}\right)}^{2}}=c\cdot \Delta t_{i}$$

where c and $\Delta t_{i}$ are known. Therefore, P(x,y,z) can be obtained by solving the following nonlinear equation:

(4) $$\{\begin{array}{c} \sqrt{{\left(x-x_{1}\right)}^{2}+{\left(y-y_{1}\right)}^{2}+{\left(z-z_{1}\right)}^{2}}-\sqrt{{\left(x-x_{0}\right)}^{2}+{\left(y-y_{0}\right)}^{2}+{\left(z-z_{0}\right)}^{2}}=c(\Delta t_{1}-\Delta t_{0})\\ \sqrt{{\left(x-x_{2}\right)}^{2}+{\left(y-y_{2}\right)}^{2}+{\left(z-z_{2}\right)}^{2}}-\sqrt{{\left(x-x_{0}\right)}^{2}+{\left(y-y_{0}\right)}^{2}+{\left(z-z_{0}\right)}^{2}}=c(\Delta t_{2}-\Delta t_{0})\\ \sqrt{{\left(x-x_{3}\right)}^{2}+{\left(y-y_{3}\right)}^{2}+{\left(z-z_{3}\right)}^{2}}-\sqrt{{\left(x-x_{0}\right)}^{2}+{\left(y-y_{0}\right)}^{2}+{\left(z-z_{0}\right)}^{2}}=c(\Delta t_{3}-\Delta t_{0}) \end{array}$$

Equation (4) is a hyperboloid locating equation. It reflects that LBL 3D positioning requires at least four hydrophones.

### 2.2. Working Principle of the System

**Figure 2:**
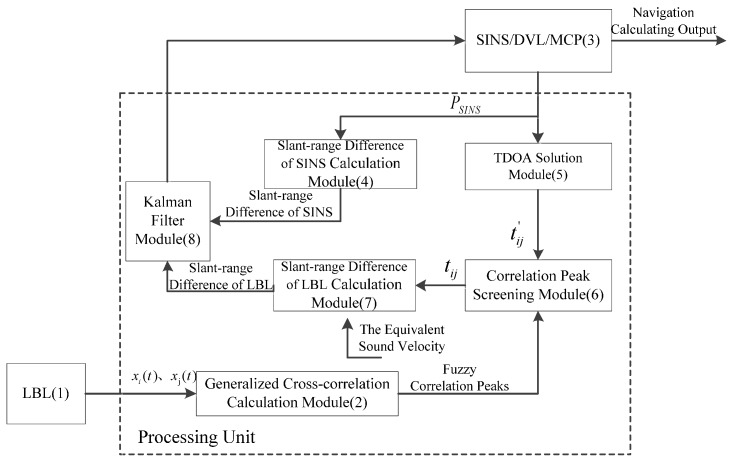
Working principle of the system.

Working principle of the system is shown in Figure 2. Firstly, hydrophones receive signals transmitted from the sound source on AUV. Generalized cross-correlation operation to signals received by hydrophones *i* and *j* ($x_{i}(t)$ and $x_{j}(t)$) was implemented. Due to refractions and reflections of sound signals during underwater propagation, a group of fuzzy correlation peaks are gained from the generalized cross-correlation operation. Next, slant-range difference between hydrophones ($\Delta R_{SINS}$) is estimated according to current AUV position information ($P_{SINS}$) of SINS/DVL/MCP system, and time difference of sound source signal arrival to hydrophone *i* and *j* ($t_{ij}^{\prime }$) is calculated. Based on $t_{ij}^{\prime }$, fuzzy correlation peaks are screened by the correlation peak screening module, getting the ideal time difference of arrival ($t_{ij}$). Later, slant-range difference based on LBL ($\Delta R_{LBL}$) is calculated from sound velocity-assisted correcting algorithm. Finally, difference between $\Delta R_{SINS}$ and $\Delta R_{LBL}$ is input into Kalman filter as an external observation variable. There are another two observation variables: velocity offered by DVL and heading angle offered by MCP. Errors of SINS are corrected based on filtering results, thus getting the final accurate AUV position ($P_{AUV}$), velocity and attitude.

## 3. TDOA Calculation Method of Underwater Acoustic Propagation Multipath

### 3.1. Fuzzy Correlation Peaks in Multipath Propagation

Underwater sound signal will make a series of refractions and reflections during propagation and signal received by hydrophones is mutual interference superposition of multipath signals. Sound signal propagation channel can be modeled as a mutual interference multipath channel [11].

**Figure 3:**
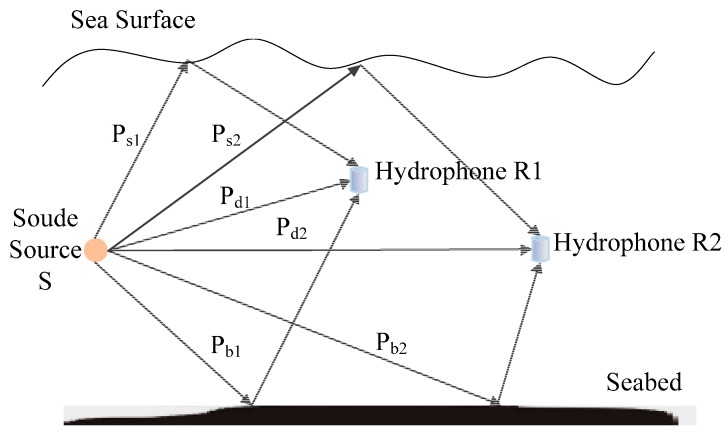
Simplified underwater multipath propagation model.

Figure 3 shows a simplified underwater sound multipath propagation model, which is composed of one sound source S and two hydrophones *R1* and *R2*. Except for the straight propagation channels *P_d1_* and *P_d2_* from sound source to hydrophones, sound signal is reflected by sea surface (*P_s1_* and *P_s2_*) and seabed (*P_b1_* and *P_b2_*) once.

**Figure 4:**
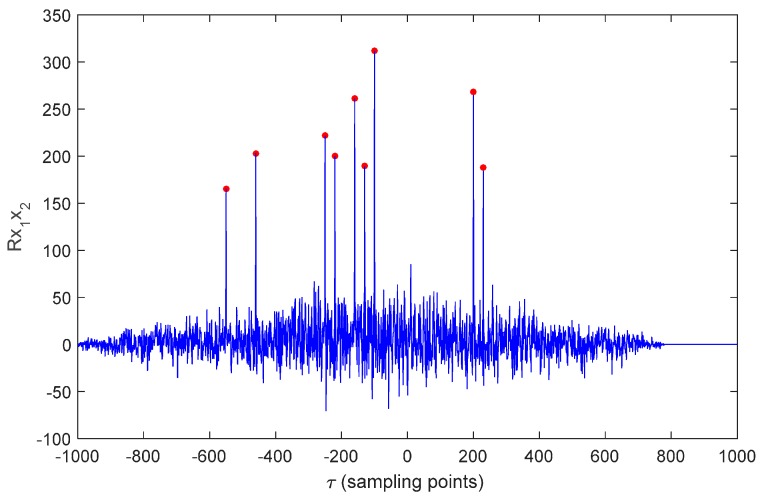
Cross-correlation function of signals.

Cross-correlation function of signals $x_{1}(t)$ and $x_{2}(t)$ received by *R1* and *R2* is shown in Figure 4. Sampling period is 5 × 10^−6^ s. Except for peaks (main peaks) at TDOA of straight paths, there are other peaks (secondary peaks). The phenomenon that there are many peaks in cross-correlation function and it is impossible to estimate TDOA of signals is called fuzzy cross-correlation peak.

### 3.2. SINS-Assisted Method to Search the Optimum TDOA

According to above analysis, multipath effect of sound signal propagation brings several correlation peaks with similar amplitudes in generalized cross-correlation operation. Due to high propagation speed of sound signal in water, tiny time difference error will cause large slant-range difference error. Using the largest peak as the main correlation peak will surely cause large errors. Therefore, it is necessary to increase calculation accuracy of ideal time difference. Combining characteristics of underwater integrated navigation system, this paper uses SINS location information to assist LBL system to search the optimum time difference under multipath propagation conditions, thus increasing positioning accuracy of the system.

In the LBL system, set $\left(x_{i},y_{i},z_{i}\right)$ be position of the *i-*th hydrophone and $\left(x_{S},y_{S},z_{S}\right)$ be the AUV position (sound source is on AUV) output by SINS. Then, distance between AUV and hydrophones is:

(5) $$R_{SINSi}=\sqrt{{\left(x_{S}-x_{i}\right)}^{2}+{\left(y_{S}-y_{i}\right)}^{2}+{\left(z_{S}-z_{i}\right)}^{2}}$$

The slant-range difference between the distances of any two hydrophones to the sound source is:

(6) $$\Delta R_{SINSij}=R_{SINSi}-R_{SINSj}(i\neq j)$$

The calculated time difference of signal arrival between two hydrophones is:

(7) $$\Delta t_{ij}^{\prime }=\frac{\Delta R_{SINSij}}{c_{ij}}$$

where $c_{ij(k)}$ is equivalent sound velocity of propagation path at the time *k* [12]. The influence factors of sound channel structure mainly include water temperature, salinity and depth. Comparing with the time *k −* 1, these factors are essentially unchanged, so the current equivalent sound velocity $c_{ij(k)}$ can be represented by the equivalent sound velocity $c_{ij(k-1)}$ of the last time cycle, which is:

(8) $$c_{ij(k)}=c_{ij(k-1)}$$

The equivalent sound velocity is the ratio of the slant-range difference between AUV which position is output by the SINS/LBL tightly coupled system and hydrophones to the time difference. The calculation process is as follows.

Set the AUV position output by the SINS/LBL tightly coupled system at time k−1 as $P_{SINS/LBL(k-1)}(x_{S/L(k-1)},y_{S/L(k-1)},z_{S/L(k-1)})$, distance between AUV and hydrophones is:

(9) $$D_{i(k-1)}=\sqrt{{\left(x_{S/L}-x_{i}\right)}^{2}+{\left(y_{S/L}-y_{i}\right)}^{2}+{\left(z_{S/L}-z_{i}\right)}^{2}}$$

The slant-range difference between AUV and any two hydrophones (*i* and *j*) is:

(10) $$\Delta D_{ij\left(k-1\right)}=D_{i\left(k-1\right)}-D_{j\left(k-1\right)}$$

If the time difference between AUV and two hydrophones at time k−1 is $\Delta t_{ij(k-1)}$, then the equivalent sound velocity of propagation path is:

(11) $$c_{ij\left(k-1\right)}=\frac{\Delta D_{ij\left(k-1\right)}}{\Delta t_{ij(k-1)}}$$

Substitute $c_{ij(k-1)}$ into Equation (7), and time difference $\Delta t_{ij}^{\prime }$ at time k can be calculated. The peak closest to $\Delta t_{ij}^{\prime }$ is found out from the fuzzy correlation peaks in Figure 4. Time difference of this peak is viewed as the ideal time difference $\Delta t_{ij}$.

## 4. SINS/LBL Tightly Coupled Model

### 4.1. Establishing LBL-Based Slant-Range Difference Model

In TDOA method, signal arrival time difference between hydrophones (1, 2, 3) and hydrophone 0 could be gained through the method in Section 3.2, thus getting the corresponding slant-range difference [9,13,14] $\Delta R_{i}^{\prime }=R_{i}-R_{0}$ (i=1,2,3):

(12) $$\left\{ \begin{array}{l} \Delta R_{i}^{\prime }=\Delta R_{i}+\delta R_{i}+\upsilon_{\Delta R_{i}}\\ \delta {\dot{R}}_{i}=-\frac{1}{\tau_{\delta R_{i}}}\delta R_{i}+\upsilon_{\delta R_{i}} \end{array}\right.$$

$\Delta R_{i}$ is truth value of slant-range difference and $\delta R_{i}$ is error of slant-range difference, which can be expressed by the first-order Gauss-Markov process. $\tau_{\delta R_{i}}$ and $\upsilon_{\delta R_{i}}$ are correlation time and driven white noise of the first-order Gauss-Markov process. $\upsilon_{\Delta R_{i}}$ is Gaussian white noise.

### 4.2. State Equation and Measurement Equation of the SINS/LBL Tightly Coupled System

(13) $$\left[\begin{array}{c} {\overset{•}{X}}_{SINS}\\ {\overset{•}{X}}_{LBL} \end{array}\right]=\left[\begin{array}{cc} F_{SINS} & 0\\ 0 & F_{LBL} \end{array}\right]\left[\begin{array}{c} X_{SINS}\\ X_{LBL} \end{array}\right]+\left[\begin{array}{c} W_{SINS}\\ W_{LBL} \end{array}\right]$$

where $X_{SINS}$ and $X_{LBL}$ are state variables of SINS and LBL; $F_{SINS}$ and $F_{LBL}$ are SINS and LBL system matrixes; and $W_{SINS}$ and $W_{LBL}$ are system noises of SINS and LBL.

Considering the environment of AUV, velocity along the height direction and location information could not be neglected. State variable of SINS is chosen as:

(14) $$\begin{array}{l} X_{SINS}=\\ {\left[\begin{array}{ccccccccccccccc} \delta V_{E} & \delta V_{N} & \delta V_{U} & \varphi_{E} & \varphi_{N} & \varphi_{U} & \delta L & \delta L & \delta h & \nabla_{bx} & \nabla_{by} & \nabla_{bz} & \varepsilon_{bx} & \varepsilon_{by} & \varepsilon_{bz} \end{array}\right]}^{T} \end{array}$$

where $\delta V_{E},\delta V_{N},\delta V_{U}$ are velocity errors of SINS toward east, north and up, respectively; $\phi_{E},\phi_{N},\phi_{U}$ are misalignment angles of SINS toward east, north and up, respectively; δL,δλ,δh are SINS latitude, longitude and height errors, respectively; $\nabla_{bx},\nabla_{by},\nabla_{bz}$ are biased errors of accelerometers along three axial directions, respectively; and $\varepsilon_{bx},\varepsilon_{by},\varepsilon_{bz}$ are three axial drifts of gyroscopes. $F_{SINS}$ can be determined by the SINS error equation.

(15) $$X_{LBL}={\left[\begin{array}{ccc} \delta R_{1} & \delta R_{2} & \delta R_{3} \end{array}\right]}^{T}$$

where $\delta R_{i}$ (i=1,2,3) is slant-range difference error.

(16) $$F_{LBL}=diag(-\frac{1}{\tau_{\delta R_{1}}},-\frac{1}{\tau_{\delta R_{2}}},-\frac{1}{\tau_{\delta R_{3}}})$$

The measurement equation of tightly coupled system is:

(17) $$Z_{SINS/LBL}=\Delta R_{SINS}-\Delta R_{LBL}=\left[\begin{array}{c} \Delta R_{SINS1}-\Delta R_{LBL1}\\ \Delta R_{SINS2}-\Delta R_{LBL2}\\ \Delta R_{SINS3}-\Delta R_{LBL3} \end{array}\right]=H_{SINS/LBL}X+\eta_{SINS/LBL}$$

where $\Delta R_{SINSi}-\Delta R_{LBLi}$ is the slant-range difference error between SINS and LBL. $\eta_{SINS/LBL}$ is measurement noise.

Let $\left(x_{S},y_{S},z_{S}\right)$ be the AUV position estimated by SINS. Then, slant-range difference is:

(18) $$\Delta R_{SINSi}=\sqrt{{\left(x_{S}-x_{i}\right)}^{2}+{\left(y_{S}-y_{i}\right)}^{2}+{\left(z_{S}-z_{i}\right)}^{2}}-\sqrt{{\left(x_{S}-x_{0}\right)}^{2}+{\left(y_{S}-y_{0}\right)}^{2}+{\left(z_{S}-z_{0}\right)}^{2}}$$

In relative to the real AUV position (x,y,z), it can get from Taylor linearization of Equation (18):

(19) $$\Delta R_{SINSi}=R_{i}-R_{0}+e_{ix}\delta x+e_{iy}\delta y+e_{iz}\delta z$$

(20) $$e_{ix}=\frac{\partial (\Delta R_{SINSi})}{\partial x}=\frac{x_{S}-x_{i}}{R_{i}}-\frac{x_{S}-x_{0}}{R_{0}}$$

(21) $$e_{iy}=\frac{\partial (\Delta R_{SINSi})}{\partial y}=\frac{y_{S}-y_{i}}{R_{i}}-\frac{y_{S}-y_{0}}{R_{0}}$$

(22) $$e_{iz}=\frac{\partial (\Delta R_{SINSi})}{\partial z}=\frac{z_{S}-z_{i}}{R_{i}}-\frac{z_{S}-z_{0}}{R_{0}}$$

where

(23) $$R_{i}=\sqrt{{\left(x_{S}-x_{i}\right)}^{2}+{\left(y_{S}-y_{i}\right)}^{2}+{\left(z_{S}-z_{i}\right)}^{2}}$$

Slant-range difference of LBL is:

(24) $$\Delta R_{LBLi}=R_{i}-R_{0}+\delta R_{i}+\nu_{\Delta R_{i}}$$

Therefore,

(25) $$\Delta R_{SINSi}-\Delta R_{LBLi}=e_{ix}\delta x+e_{iy}\delta y+e_{iz}\delta z-\delta R_{i}-\nu_{\Delta R_{i}}$$

δx,δy,δz in Equation (25) are expressed by δL,δλ,δh:

(26) $$\left\{ \begin{array}{l} \delta x=\delta h\text{cos}L\text{cos}\lambda -(R_{E}+h)\sin L\text{cos}\lambda \delta L-(R_{E}+h)\text{cos}L\sin\lambda \delta \lambda \\ \delta y=\delta h\text{cos}L\sin\lambda -(R_{E}+h)\sin L\sin\lambda \delta L-(R_{E}+h)\text{cos}L\text{cos}\lambda \delta \lambda \\ \delta z=\delta h\sin L+\left[R_{E}(1-e^{2})+h\right]\text{cos}L\delta L \end{array}\right.$$

Substitute Equation (26) into Equation (25) and the measurement matrix in Equation (17) could be gained:

(27) $$H_{SINS/LBL}=\left[\begin{array}{ccccc} 0_{3\times 6} & H_{1(3\times 3)} & 0_{3\times 6} & -E_{3\times 3} & 0_{3\times 5} \end{array}\right]$$

where

(28) $$H_{1}=\left[\begin{array}{ccc} a_{11} & a_{12} & a_{13}\\ a_{21} & a_{22} & a_{23}\\ a_{31} & a_{32} & a_{33} \end{array}\right]$$

(29) $$E_{3\times 3}=\left[\begin{array}{ccc} 1 & 0 & 0\\ 0 & 1 & 0\\ 0 & 0 & 1 \end{array}\right]$$

Elements in $H_{1}$ are:

(30) $$\begin{array}{lll} a_{i1} & = & -\left(R_{E}+h\right)\sin L\text{cos}\lambda e_{ix}-\left(R_{E}+h\right)\sin L\sin\lambda e_{iy}\\ & & +[R_{E}(1-e^{2})+h]\text{cos}Le_{iz} \end{array}$$

(31) $$a_{i2}=-\left(R_{E}+h\right)\text{cos}L\sin\lambda e_{ix}-\left(R_{E}+h\right)\text{cos}L\text{cos}\lambda e_{iy}$$

(32) $$a_{i3}=\text{cos}L\text{cos}\lambda e_{ix}+\text{cos}L\sin\lambda e_{iy}+\sin Le_{iz}$$

where i(i=1,2,3), $R_{E}$ is radius of curvature in the normal plane perpendicular to meridian plane; and e is ovality of the ellipsoid.

## 5. SINS/DVL/MCP/LBL Integration System

**Figure 5:**
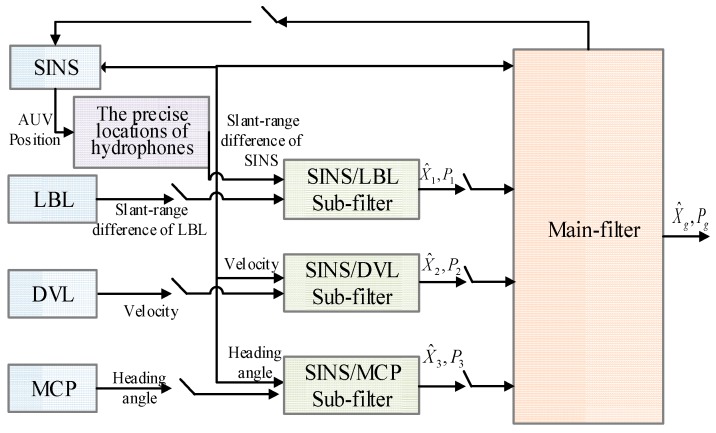
The federal system based on tightly coupled SINS (Strapdown Inertial Navigation System)/LBL (Long Base Line).

Figure 5 is federated Kalman filter structure of underwater integrated navigation system. It contains SINS/LBL tightly coupled sub-system, SINS/DVL sub-system and SINS/MCP sub-system.

### 5.1. SINS/LBL Tightly Coupled Sub-System

State equation and measurement equation of SINS/LBL tightly coupled sub-system are introduced in Section 4.2.

### 5.2. SINS/DVL Sub-System

(1) State equation of SINS/DVL sub-system is described as:

(33) $$\left[\begin{array}{c} {\overset{•}{X}}_{SINS}\\ {\overset{•}{X}}_{DVL} \end{array}\right]=\left[\begin{array}{cc} F_{SINS} & 0\\ 0 & F_{DVL} \end{array}\right]\left[\begin{array}{c} X_{SINS}\\ X_{DVL} \end{array}\right]+\left[\begin{array}{c} W_{SINS}\\ W_{DVL} \end{array}\right]$$

where the state vector of SINS ($X_{SINS}$) and system matrix $F_{SINS}$ are same as those in Section 4.2. $W_{SINS},W_{DVL}$ are system noises of SINS and DVL, respectively.

(34) $$X_{DVL}=\left[\begin{array}{ccc} \delta V_{Dx} & \delta V_{Dy} & \delta V_{Dz} \end{array}\right]$$

where $\delta V_{Di}(i=x,y,z)$ is velocity measurement error caused by topographical changes, which could be described approximately by the first-order Gauss-Markov process:

(35) $$F_{DVL}=diag(-\frac{1}{\tau_{\delta dx}},-\frac{1}{\tau_{\delta dy}},-\frac{1}{\tau_{\delta dz}})$$

where $\tau_{\delta di}(i=x,y,z)$ is correlation time of the first-order Gauss-Markov process.

(2) Measurement equation of SINS/DVL sub-system:

(36) $$Z_{SINS/DVL}=H_{SINS/DVL}\left[\begin{array}{c} X_{SINS}\\ X_{DVL} \end{array}\right]+\eta_{SINS/DVL}$$

where $H_{SINS/DVL}$ is measurement matrix of SINS/DVL sub-system and $\eta_{SINS/DVL}$ is measurement noise.

Velocity difference between SINS and DVL is taken as the observation variable. Since DVL measures AUV speed in the carrier coordinate system directly, it shall transform output velocity of DVL into geographic coordinate system.

(37) $$Z_{SINS/DVL}=(V_{SINS}^{n}+\delta V_{SINS}^{n})-C_{b}^{n}(V_{DVL}^{b}+\delta V_{DVL}^{b})=\delta V^{n}-V^{n}\times \varphi -C_{b}^{n}V_{DVL}^{b}$$

where $V_{SINS}^{n}$ and $\delta V_{SINS}^{n}$ are truth value and error of SINS velocity in geographic coordinate system, respectively; $V_{DVL}^{b}$ and $\delta V_{DVL}^{b}$ are truth value and error of DVL velocity in carrier coordinate system, respectively; $V^{n}$ is AUV speed; φ is misalignment angle; and $C_{b}^{n}$ is attitude transfer matrix from carrier coordinate to geographic coordinate.

(38) $$Z_{SINS/DVL}=\left[\begin{array}{cccccc} 1 & 0 & 0 & 0 & V_{U} & -V_{N}\\ 0 & 1 & 0 & -V_{U} & 0 & V_{E}\\ 0 & 0 & 1 & V_{N} & -V_{E} & 0 \end{array}0_{3\times 9}\begin{array}{ccc} -C_{11} & -C_{12} & -C_{13}\\ -C_{21} & -C_{22} & -C_{23}\\ -C_{31} & -C_{32} & -C_{33} \end{array}\right]\left[\begin{array}{c} X_{SINS}\\ X_{DVL} \end{array}\right]+\eta_{SINS/DVL}$$

where $V_{E},V_{N},V_{U}$ are velocity of AUV toward east, north and up direction, respectively. $C_{ij}(i=1,2,3;j=1,2,3)$ are elements in the attitude transfer matrix $C_{n}^{b}$.

### 5.3. SINS/MCP Sub-System

State equation of SINS/MCP sub-system is described as:

(39) $$\left[\begin{array}{c} {\overset{•}{X}}_{SINS}\\ {\overset{•}{X}}_{MCP} \end{array}\right]=\left[\begin{array}{cc} F_{SINS} & 0\\ 0 & F_{MCP} \end{array}\right]\left[\begin{array}{c} X_{SINS}\\ X_{MCP} \end{array}\right]+\left[\begin{array}{c} W_{SINS}\\ W_{MCP} \end{array}\right]$$

State sector of SINS ($X_{SINS}$) and system matrix $F_{SINS}$ are same as those in Section 4.2.

Magnetic compass uses only one state variable—heading angle error:

(40) $$X_{MCP}=\delta \psi_{MCP}$$

$\delta \psi_{MCP}$ can be set a random constant. Therefore,

(41) $$F_{MCP}=O_{1\times 1}$$

Heading angle difference between SINS and MCP is used as the observation variable, and the measurement equation of SINS/MCP sub-system is:

(42) $$Z_{SINS/MCP}={\hat{\psi }}_{SINS}-{\hat{\psi }}_{MCP}=H_{SINS/MCP}\left[\begin{array}{c} X_{SINS}\\ X_{MCP} \end{array}\right]+\eta_{SINS/MCP}$$

where $H_{SINS/MCP}$ is measurement matrix of SINS/MCP sub-system, $H_{SINS/MCP}=\left[\begin{array}{cccc} 0_{1\times 5} & 1 & 0_{1\times 15} & -1 \end{array}\right]$ and $\eta_{SINS/MCP}$ is measurement noise.

## 6. Simulation and Experiment

### 6.1. Simulation of SINS-Assisted LBL Tracking Optimum Time Difference under Static Conditions

**Figure 6:**
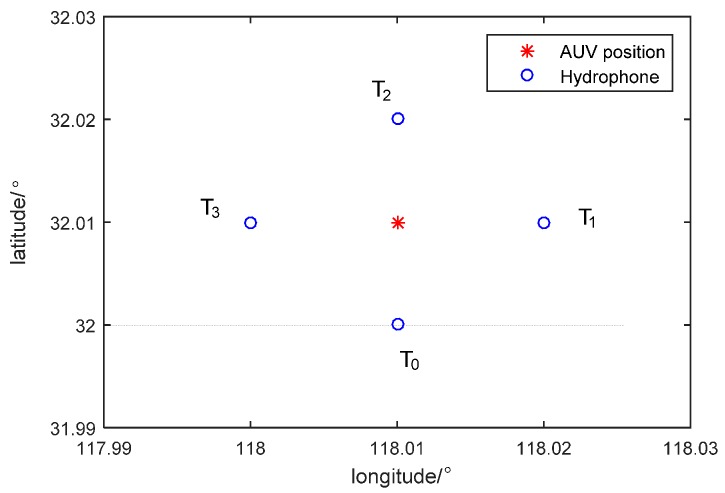
Layout of hydrophones and AUV (Autonomous Underwater Vehicle) position.

Hydrophones and sound source layouts are shown in Figure 6. Longitudes and latitudes of four hydrophones are (118.01°, 32°), (118.01°, 32.02°), (118.02°, 32.01°) and (118°, 32.01°), respectively. They are all places at a depth of 30 m. Position coordinates of sound source is (118.01°, 32.0925°) and its depth is 10 m.

**Figure 7:**
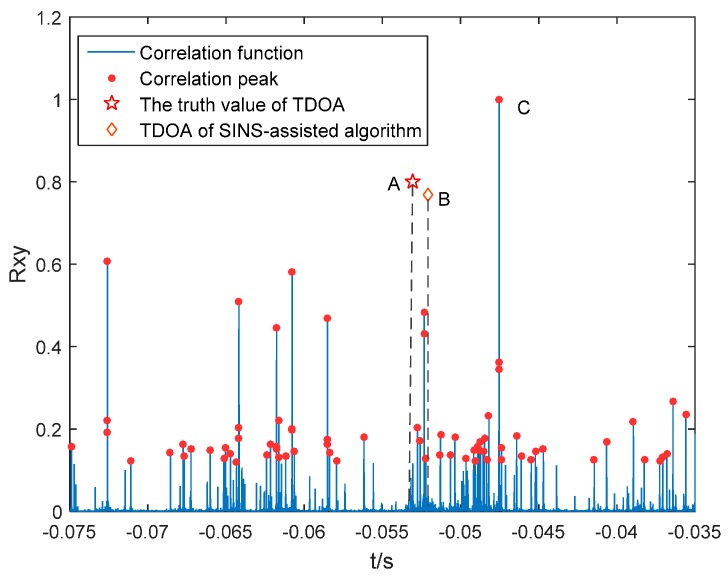
Screening of main correlation peak.

The cross-correlation function curves of received signals by hydrophones *T_1_* and *T_0_* are show in Figure 7 Due to multiple reflections of sound signals underwater, many correlation peaks are gained from cross-correlation operation. Many correlation peaks have similar amplitude, which makes it difficult to determine which peak shows the correct time difference.

Since sound signal is reflected many times during underwater propagation, the generalized cross-correlation operation will bring many correlation peaks. In traditional algorithms, time difference corresponding to the correlation peak with the largest generalized cross-correlation function value (Point C in Figure 7) is used as TDOA of different hydrophones. It reveals from Figure 7 that TDOA with traditional method differs from truth value (Point A in Figure 7, red star) significantly, while TDOA with SINS aiding method (Point B in Figure 7, red diamond) is close to the true time difference. Thus, choosing the peak nearest to point B could get the minimum time difference error. The proposed algorithm selects the ideal time difference according to the position output by SINS/DVL/MCP system [12]. Comparisons between this improved algorithm and the traditional algorithm are shown in Table 1 and Table 2. The improved algorithm solves interference of multipath effect to the optimum TDOA estimation and has higher TDOA calculation accuracy than the traditional algorithm.

### 6.2. Simulation of SINS/LBL Tightly Coupled Algorithm

**Figure 8:**
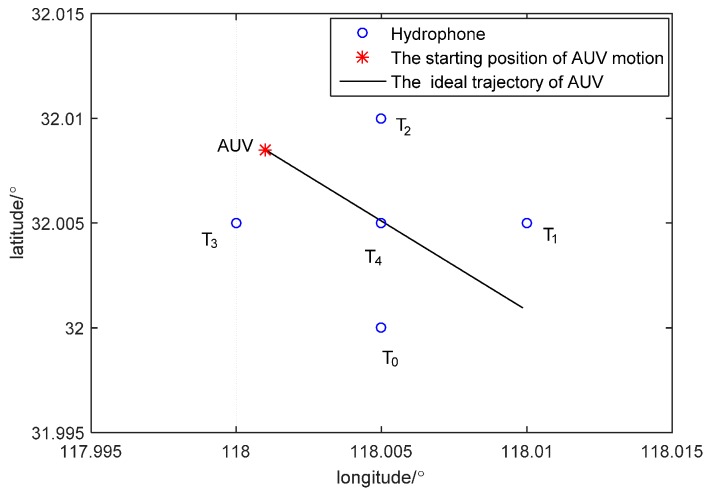
Hydrophone layout and initial position of AUV.

**Figure 9:**
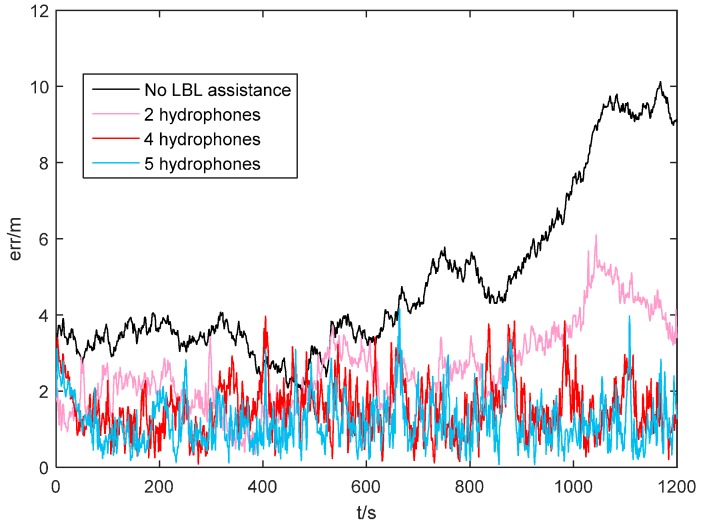
Errors of SINS/LBL tightly coupled algorithm.

To study effectiveness of the proposed SINS/LBL tightly coupled algorithm and effect of the number of available hydrophones on positioning accuracy, simulation experiments of SINS combining with 5, 4 and 2 hydrophones are carried out. In Figure 8, five hydrophones are installed at *T*_0_(118.005°, 32.005°), *T*_1_(118.005°, 32°), *T*_2_(118.01°, 32.005°), *T*_3_(118°, 32.01°) and *T*_4_(118.005°, 32.01°), at a depth of 30 m. AUV depth is 10 m. From the initial position (118.001°, 32.0085°), AUV travels at a constant speed (1 m/s) along 135° north by east. The random drift and constant drift of the gyroscope are both 0.04 °/h. Random drift and constant drift of the accelerometer are 50μg and 100μg, respectively. The initial misalignment angles are: 0.01° pitching angle, 0.01° angle of roll and 0.1° heading angle. DVL velocity error is 0.1 m/s. Heading angle error of MCP is 0.3° and the simulation time is 1200 s. In the simulation, the number of hydrophones is set through software. When SINS combines with five hydrophones, it uses *T_0_*, *T_1_*, *T_2_*, *T_3_* and *T_4_*. When SINS combines with four hydrophones, it uses *T_0_*, *T_1_*, *T_2_* and *T_3_*. When SINS combines with two hydrophones, it only uses *T_2_* and *T_3_*. Simulation results are shown in Figure 9 and Table 3.

It can be seen in Figure 9 and Table 3 that when four hydrophones are installed at seabed, LBL provides three slant-range differences to form a tightly coupled system with SINS. Position error of SINS/LBL tightly coupled system is small (<4 m) and convergent. At this moment, navigation performance is satisfying. When there are five hydrophones, position error is almost equal to that when there are four hydrophones, indicating that in this situation increase of hydrophones does not improve positioning accuracy significantly. When there are two hydrophones, LBL provides only one slant-range difference for SINS. Position error is large and fluctuates violently. However, it could still compensate for position error compared to the system without hydrophones. This technology could ensure that AUV can compensate for position error quickly when it just enters into the LBL array.

### 6.3. Simulation of AUV Dynamic Positioning Based on Federated Filter System

Simulation of AUV dynamic positioning was implemented in order to further verify effectiveness of SINS/LBL tightly coupled algorithm. Four hydrophones are installed at seabed. They are put at *T*_0_(118°, 32.01°), *T*_1_(118.01°, 32°), *T*_2_(118.02°, 32.01°) and *T*_3_(118.01°, 32.02°) at a depth of 30 m. Suppose AUV travels from the initial position (117.98°, 32.036°) at a constant speed (1 m/s) along 135° north by east. Rest simulation conditions are same as above and simulation time is set 3 h.

**Figure 10:**
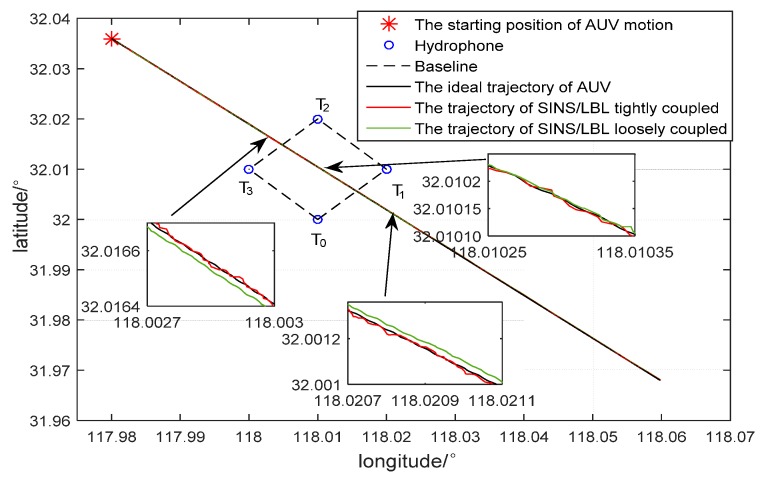
Simulation of SINS/LBL dynamic path.

**Figure 11:**
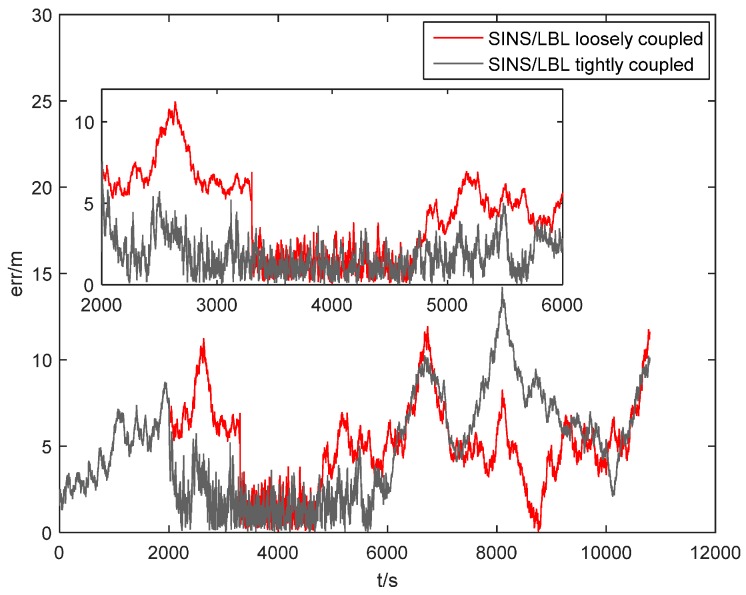
Position error comparison of SINS/LBL dynamics simulations.

In the simulation, transmission range of sound source is 1.5 km. From 2000~3300 s, AUV approaches the hydrophone array, so *T_2_* and *T_3_* could receive sound signal. From 3300~4700 s, AUV enters into the hydrophone array and all four hydrophones could receive sound signal. From 4700~6000 s, AUV departs away from the hydrophone array gradually, when only *T_0_* and *T_1_* could receive sound signal. After 6000 s, AUV leaves. Simulation results are shown in Figure 10 and Figure 11.

Figure 10 is SINS/LBL dynamic simulation results. When AUV approaches the hydrophone array, movement track of SINS/LBL tightly coupled system deviates from the real track slightly, while movement track of SINS/LBL loosely coupled system deviates significantly. When AUV is at center of the hydrophone array, movement tracks of both SINS/LBL tightly and loosely coupled systems are similar with the real track. When AUV begins to depart away from the hydrophone array, movement track of SINS/LBL tightly coupled system keeps a small error with the real track for a while, while movement track of the SINS/LBL loosely coupled system deviates from the real track gradually.

Figure 11 shows position error comparison of SINS/LBL dynamics simulations. It reflects position errors of two algorithms during the whole process intuitively. From 0~2000 s, AUV is far away from the hydrophone array. During this period, it is impossible to use LBL to compensate position error and the system only involves SINS/DVL/MCP. As a result, position error accumulates gradually, reaching 8 m. From 2000~3300 s, AUV approaches the signal area of the hydrophone array gradually. *T_2_* and *T_3_* provide one slant-range difference for SINS, which corrects position error of AUV to a certain extent. Position error decreases to 5 m. Since SINS/LBL loosely coupled algorithm requires at least four hydrophones, it is invalid during this period and position error continues to accumulate, reaching 10 m. From 3300~4700 s, AUV enters into the hydrophone array completely and all four hydrophones could receive sound signals. LBL provides three slant-range differences, which reduces position error quickly. Meanwhile, loosely coupled algorithm is effective and inhibits divergence of position error. Therefore, both two algorithms achieve equivalent position error accuracy, less than 4 m. From 4700~6000 s, AUV departs away from the hydrophone array and only *T_0_* and *T_1_* could receive sound signals. Although LBL provides only one slant-range difference, the SINS/LBL integrated algorithm still could correct position error to a certain extent. Position error is controlled within 5 m. During this period, loosely coupled algorithm could not work normally and has the larger position error (7 m). After 6000 s, AUV leaves the hydrophone array completely and position information is provided by SINS/DVL/MCP. It reveals that there is still effective compensation of position error.

## 7. Conclusions

This paper puts forward a SINS/LBL tightly coupled navigation system to solve position error accumulation of AUV. It uses slant-range difference as an observation variable for filtering correction of system error. Moreover, to solve fuzzy correlation peaks caused by underwater propagation multipath of LBL sound signal, a SINS-assisted LBL method to search the optimal TDOA is proposed. This method is effective and easy to operate. Test results demonstrate that the SINS/LBL tightly coupled system is more reliable than SINS/LBL loosely coupled system and could still correct position error when there are fewer than four available hydrophones. It expands navigational area of AUV and makes navigation track more flexible.

All simulations in this paper assume that undersea hydrophones are laid in a diamond shape and the hydrophone line up is single, and the next step could be studying different ways hydrophones line up to verify the effectiveness of the SINS/LBL tightly coupled algorithm. Due to the limitations of experimental conditions, the algorithm has not yet been verified in actual conditions. If the conditions are met, we will do further work in the future.

## Figures and Tables

**Table 1:** Comparison of TDOA (Time Difference of Arrival) calculation error.

TDOA of Two Hydrophones	Error of Traditional Algorithm/s	Error of SINS-Assisted Algorithm/s
$t_{T_{1}}-t_{T_{0}}$	0.0056	0.0010
$t_{T_{2}}-t_{T_{0}}$	0.0055	−0.0009
$t_{T_{3}}-t_{T_{0}}$	0.0034	0.0009

Note: $t_{T_{i}}-t_{T_{0}},(i=1,2,3)$ is TDOA between hydrophone *T_i_* and hydrophone *T_0_*.

**Table 2:** Comparison of slant-range difference calculation error.

Slant-Range Difference between Two Hydrophones and Sound Source	Error of Traditional Algorithm/m	Error of SINS-Assisted Algorithm/m
$R_{T_{1}}-R_{T_{0}}$	8.0626	1.4379
$R_{T_{2}}-R_{T_{0}}$	8.8442	−1.2836
$R_{T_{3}}-R_{T_{0}}$	5.0872	1.3466

Note: In Table 2, $R_{T_{i}}-R_{T_{0}},(i=1,2,3)$ is difference between sound source distance to the hydrophone *T_i_* and sound source distance to the hydrophone *T_0_*.

**Table 3:** Position errors of SINS/LBL tightly coupled algorithm.

	No LBL Assistance	2 Hydrophones	4 Hydrophones	5 Hydrophones
Mean /m	4.8300	2.5760	1.5420	1.1900
Variance /m	2.1890	1.1200	0.7039	0.6336
